# Chemical profiling, in vitro antioxidant, membrane stabilizing and antimicrobial properties of wild growing *Murraya paniculata* from Amarkantak (M.P.)

**DOI:** 10.1038/s41598-021-87404-7

**Published:** 2021-05-07

**Authors:** Shruti Sonter, Shringika Mishra, Manish Kumar Dwivedi, Prashant Kumar Singh

**Affiliations:** Department of Biotechnology, Indira Gandhi National Tribal University, Amarkantak, Anuppur, Madhya Pradesh 484887 India

**Keywords:** Drug discovery, Microbiology

## Abstract

The excessive usage of antibiotics in humans and veterinary medicine has lead to the emergence of antibiotic resistance and now requires the use of novel antibiotics. There has been increased interest towards plants as source of drugs because of their pharmacological potency and long traditional usage. The aim of the current study was to evaluate bioactive components, antioxidant, and anti-inflammatory activities of the leaf extracts of *Murraya paniculata,* a plant traditionally used in Indian medicinal system. Evaluations were made for phytochemical analysis, antioxidant, membrane stabilizing, and antimicrobial activities. The methanol extract displayed the highest flavonoid and phenolic content, the acetone extract demonstrated considerable ABTS inhibitory activity (IC_50_value:555.18 ± 1.68 µg/mL) and the hexane extract exhibited highest H_2_O_2_ radical scavenging activity (IC_50_value: 509.84 ± 3.03 µg/mL). The aqueous extract displayed 19.4 ± 0.66% RBC hemolysis and 80.5 ± 0.66% protection caused by hypotonic solution at high concentration of the extract. The fractions of hexane extract revealed a higher zone of inhibition than crude extract. The major components found in the fractions were cyclohexane (40.11%) and 3-(6-Methoxy-3-methyl-2-benzofuranyl) Cyclohexanone (13.68%) as analyzed by GC–MS/MS technique. The current results validate the traditional use of the *M. paniculata* and warrant its potential in drug development programs in further investigations.

## Introduction

The discovery of compounds with antimicrobial properties has revolutionized modern medicine systems. Use of antibiotics has provided successful interventions in surgical procedures, organ transplants, and patient care. However,reports of antimicrobial resistance amongst common bacterial pathogens are now threatening this therapeutic aid^1^. A large number of diseases such as pneumonia, tuberculosis, gonorrhoea, and salmonellosis are becoming harder to treat with the currently available antibiotics. It has been estimated that around 300 million premature deaths will occur by 2050 with a global economic loss of up to $100 trillion^2^. The resistance against antibiotics is an immediate threat to the treatment of infectious diseases as the development of new antimicrobial agents is declining and very few drugs have been approved in the recent years^3^. Phytochemicals have displayed potential antibacterial properties against sensitive and resistant pathogens. Thus,it is a priority in medical research to screen plant extracts for novel antimicrobial properties as a possible source for a new drug development^4, 5^. Plants possess a variety of secondary metabolites that can be utilized for the development of biocompatible therapeutics. Many traditional medicinal systems practiced in major parts of the world are based largely on therapeutic use of such medicinal plants. The World Health Organization (WHO) has also emphasized that several countries are gradually accepting the contribution of traditional medicine to the health and well-being of individuals, and the need to integrate it to their healthcare systems^6^. Thus, a logical application is that plants be selected based on traditional medicinal knowledge and screened for characterization of natural products as novel antimicrobials. Free radicals play a central role in various physiological conditions and have been linked to a majority of diseases and disorders^7^. Free radicals can be generated in living organisms as a normal part of metabolism by oxidation–reduction reactions catalyzed by enzymes such as catalase and peroxidase or due to exposure of biological systems to external factors such as chemical carcinogens or ionizing radiation. Reactive oxygen species (ROS) and reactive nitrogen species (RNS) may cause disintegration of cell membranes or adversely affect nucleic acids, lipids, and proteins, thereby altering the normal redox status leading to increased oxidative stress^8^. This results in development of various diseases such as cancer, arteriosclerosis, diabetes mellitus, Alzheimer’s disease, liver and skin damage, coronary heart diseases, arthritis, and inflammation^9^.

Inflammation is a non-specific response employed by both innate and adaptive immune systems against pathogenic intruders, hazardous stimuli such as allergens or tissue injury. Unregulated inflammatory responses are the underlying cause of several disorders including allergies, cardiovascular dysfunctions, metabolic syndrome, cancer, and autoimmune diseases^10^. Synthetic chemical drugs, including steroids, non-steroid anti-inflammatory drugs, and immunosuppressant, are used for regulating inflammatory crises; however, there are associated adverse effects related to gastrointestinal, cardiovascular and kidney functions^11^. Alternatively, natural compounds, dietary supplement, and herbal remedies are known to be beneficial and are gaining importance for the prevention and treatment of inflammatory diseases. Some medicinal plants which have anti-inflammatory properties associated with the presence of naturally occurring antioxidants, such as polyphenols, carotenoids, flavonoids, ascorbic acid, and tocopherols, have been reviewed thoroughly^12–14^. Vitamins A, C, and E are known to terminate lipid peroxidation chain reactions, and bioflavonoids that are widely distributed in fruits and vegetables function by scavenging the free radicals;exerting a protective effect on DNA damage possibly by chelating metal ions. Carotenoids, the most common lipid soluble phytonutrient, protect cellular membranes and lipoproteins against the ROS by scavenging peroxyl radicals^15,16^.

One plant of interest in the search for plant-based therapeutics and antimicrobial compounds is *Murraya* *paniculata* (also known as *Cholcas paniculata* L, *Chalcas exotica* L, and *Murraya exotica* L.).Commonly known as Orange Jasmine, mock orange, Chinese box, or *Kamini*, is a tropical, evergreen plant with tiny, white, scented flowers, and is cultivated as an ornamental tree or hedge^17^. The leaves have been used as a food additive in many Indian and Malay dishes due to their strong fragrance. The plant is known for its therapeutic usage and has been traditionally used for the treatment of tuberculosis, diarrhea, abdominal pain, dysentery, headache, edema, thrombosis, and stasis of blood. More than 120 secondary metabolites have been reported from *M. paniculata*, polymethoxylated flavonoids and their glycosides being the prominent ones^18,19^. The plant is also known to contain coumarins^20,21^ indole alkaloids^22–24^, and essential oils^25–27^. *M. paniculata* has been reported to display several ethno-pharmacological activities such as anti-anxiety, anti-depression^28^ antioxidant^17,29–31^, antimicrobial^30,32–35^, anti-inflammatory, antifungal^36–38^, antifeedent, toothache ^39^, diarrhea, asthama and hypertension^40^, nematicidal and toxicity analysis^41,42^ diabetic nephropathy and cardiomyopathy^43,44^. Traditional medicinal healers of Amarkantak region utilize medicinal plants for the treatment of various diseases, including *M. paniculata*. In our initial studies we found *M. paniculata* to be one such promising plant used by the healers for the successful treatment of wounds, tuberculosis, malaria, and as an antidote for snakebite. The different parts of the plant such as leaves, roots, and stem are used in the preparation of herbal remedies. To the best of our knowledge, there are no reports of systematic validation of the medicinal use of *M. paniculata* in traditional medicine. Thus, it was critical to define its pharmacological properties, such as antioxidant, antimicrobial and anti-inflammatory capabilities. The present study was undertaken to compare efficiency of different leaf extracts to find out the phyto-constituents, total phenolic, total flavonoid content, total reducing power and antimicrobial property against selected microbial strains. To our knowledge, this is the first reporting of the comparative account of the pharmacological efficiency of different leaf extracts of *M. paniculata.*

## Results

### Extract preparation

The extracts were prepared using dried leaves (100 g) of the plant in different solvents such as hexane, acetone, chloroform, methanol, and water. The results display that maximum yield per gram is obtained from methanol (4.8 g) followed by acetone (3.7 g), water (3.47 g), hexane (1.78 g), and chloroform (1.07 g). These data highlight methanol as an efficient solvent for extraction of phytochemicals from leaves of *M. paniculata.*

### Phytoconstituents analysis

Alkaloids, flavonoids, steroids, and terpenoids were detected as major secondary metabolites in the plant extracts. Tannins, saponins and glycosides were present in minor quantities while phlobatanins and terpenoids were not present in any of the extracts (Table S1).

### Total phenolic and total flavonoid content

The total phenolic and total flavonoid content in the different extracts of *M. paniculata* is represented in Table 1. Among the five extracts, methanol extract possesses highest phenolic content (1060 ± 52.83 mg gallic acid equivalent/g dry material) followed by acetone (849.8 ± 49.2 mg/g), water (114.4 ± 10.49 mg/g), chloroform (21.32 ± 3.6 mg/g), and hexane (22.17 ± 5.5 mg/g). The total flavonoid concentration in different solvent extracts was estimated using quercetin as a standard and values expressed in mg quercetin equivalent /g dry wt. Out of five extracts the methanol (318.4 ± 9.16 mg) contains highest flavonoid content followed by water (244.8 ± 7.98 mg), acetone (121.8 ± 4.42 mg), hexane (43.75 ± 1.5 mg), and chloroform (42.83 ± 6.66 mg).

**Table 1:** Quantification of total Phenolic and Flavonoid contents in *M. paniculata* leaves extracts.

*M. paniculata* Extracts	TPC^1^ (mg Gallic acid Equivalent/g dry material)	TFC^2^ (mg Quercetine Equivalent/g dry material)
MPH	20.62 ± 4.11	43.75 ± 1.528
MPA	849.8 ± 49.26^b^	121.8 ± 4.42 ^c^
MPC	21.32 ± 3.67	42.83 ± 6.66
MPM	1060 ± 52.83^b^	318.4 ± 9.16 ^a^
MPW	114.4 ± 10.49	244.8 ± 7.98 ^b^

^1^Total phenolic content and ^2^Total flavonoids content expressed as Mean ± SE (n = 3). The superscripted alphabets represent the number of extracts over which the values of the given extract are very highly significant (***). ^a^: four extracts, ^b^: three extracts, ^c^: two extracts.

### Antioxidant activity

Supplementary figure (Fig. S1) shows the concentrations of plant extract with percentage inhibition of ABTS radical. The acetone extract demonstrated elevated ABTS^+^ inhibition with increased concentration. For ABTS^+^ inhibition the IC_50_ valuesranged between 500–800 µg/mL (Table 2). The IC_50_value of acetone (555.18 ± 1.68 µg/mL) were less than the standard (ascorbic acid) used. The percentage of H_2_O_2_ inhibition by different extracts was found to be concentration dependent (Fig. S2). The hexane extracts exhibited highest H_2_O_2_ scavenging ability compared to other extracts. The IC_50_ values ranged between 500–800 µg/mL (Table 2). The IC_50_value of hexane (509.84 ± 3.03 µg/mL) was lowest as compared to the standard (ascorbic acid) or other extracts that were analyzed. These result demonstrate that hexane and acetone have better antioxidant property as compared to the standard.

**Table 2:** Free radical scavenging ability of *M. paniculata* extracts from ABTS and H_2_O_2_ scavenging assays, FRAP values represents as equivalent of Fe2 + /gram sample.

Sample	ABTS		H2O2		FRAP value Mean ± SE
	IC50 value (µg/ml) Mean ± SE	R2	IC50 value (µg/ml) Mean ± SE	R2	
MPH	854.00 ± 1.59***	0.995	509.84 ± 3.03***	0.982	0.011 ± 0.001
MPA	555.18 ± 1.68***	0.989	812.65 ± 22.29**	0.965	0.051 ± 0.003
MPM	684.39 ± 3.68**	0.972	639.26 ± 11.84***	0.986	0.085 ± 0.002
MPC	786.29 ± 3.16***	0.986	594.47 ± 0.92***	0.982	0.020 ± 0.000
MPW	814.82 ± 6.27***	0.992	732.80 ± 7.9**	0.932	0.046 ± 0.002
AA	664.90 ± 2.81***	0.992	528.01 ± 4.37***	0.983	0.223 ± 0.002

Values represented as Mean ± SE (n = 3). *P value* (***): very highly significant; (**): highly significant, (*): significant.

**Figure 1:**
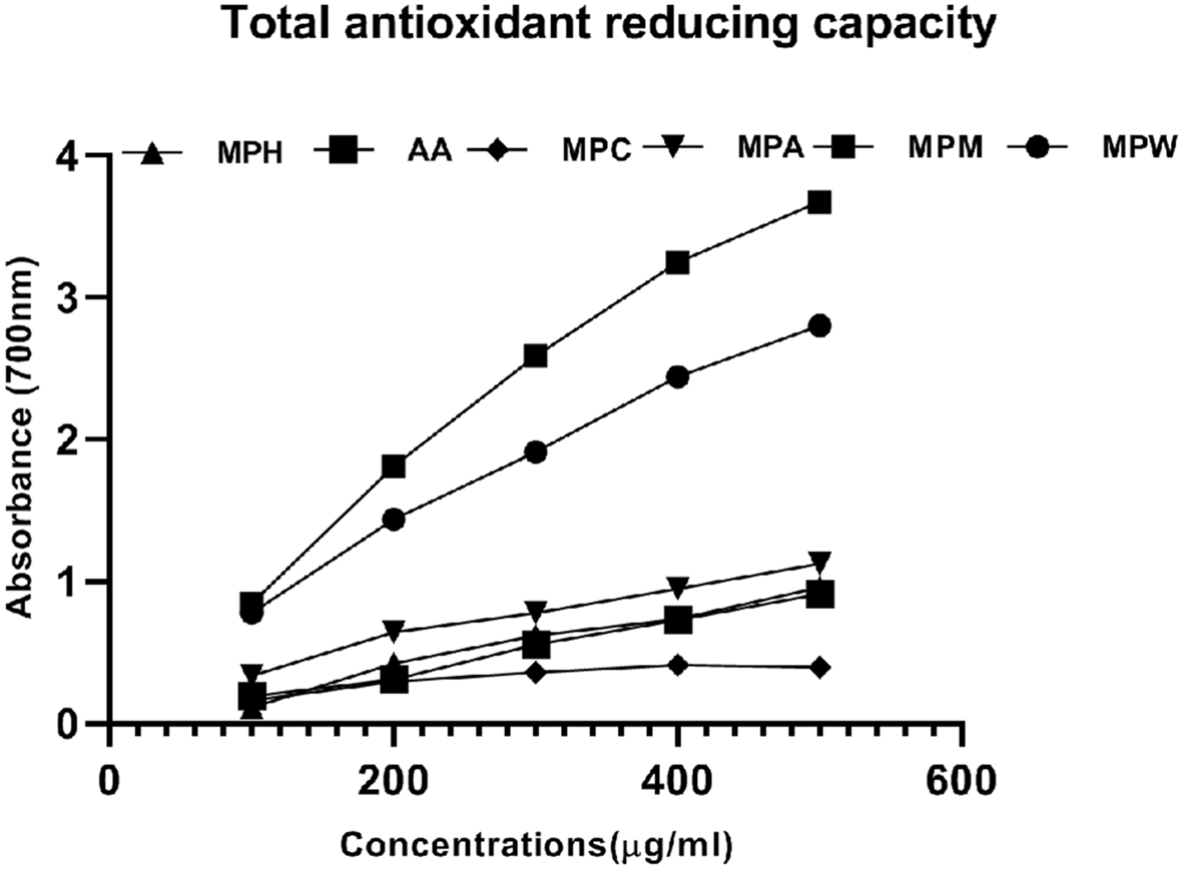
Total antioxidant reducing capacity of different concentrations of M. *paniculata* leaf extracts by reducing power assay. MPH: *M. paniculata* Hexane, MPA: *M. paniculata* Acetone, MPM: *M. paniculata* Methanol, MPC: *M. paniculata* chloroform, MPW: *M. paniculata* water and AA: Ascorbic acid.

The analysis of total reducing capacity of extracts was carried out using FRAP and reducing power assays. FRAP value of methanolic extract (0.085 ± 0.002 Fe2+/gram sample) showed highest value, followed by acetone, water, chloroform, and hexane (Table 2) The reducing power property of compound/extracts indicates its electron donating capacity. In reducing power assay, the *M. paniculata* methanol extract displayed maximum absorbance followed by water, acetone, ascorbic acid, hexane, and chloroform extracts (Fig. 1).

### Membrane stabilizing property

The results of membrane stabilization assays are presented in Table 3. It was observed that all the extracts of *M. paniculata* exhibited concentration-dependent anti-inflammatory properties as compared to the standard drug, indomethacin. The maximum concentration analyzed was 5000 µg/mL and extracts were found to possess anti-inflammatory properties in the order MPW > MPM > MPH > MPA > MPC. The aqueous extract showed the 80% protection, whereas the standard drug showed 83% protection at highest concentration tested. Pearson’s correlation was done with concentration against percent protection provided by different extracts (Table S2), where we found strong positive correlation with concentration and percent protection with all extracts.

**Table 3:** Effect of *M. paniculata* extracts and Indomethacin on HRBC membrane hemolysis and protection.

Conc^**n**^ (µ/ml)	% Hemolysis					
	MPH	MPA	MPC	MPM	MPW	INDO
1000	85.80 ± 0.55	77.36 ± 0.18	92.34 ± 0.34	59.36 ± 0.78	91.9 ± 0.67	85.96 ± 0.57
2000	50.73 ± 0.79	66.38 ± 0.40	86.59 ± 0.50	48.34 ± 2.51	73.7 ± 1.08	72.21 ± 0.36
3000	45.53 ± 0.34	48.04 ± 0.34	81.51 ± 0.67	43.15 ± 0.69	40 ± 1.34	53.31 ± 0.42
4000	40.04 ± 0.49	46.69 ± 0.47	77.03 ± 0.72	37.81 ± 1.12	32 ± 1.49	36.05 ± 0.49
5000	34.85 ± 0.10	40.12 ± 0.52	73.37 ± 0.69	29.06 ± 3.06	19.5 ± 0.38	16.7 ± 0.46

Conc^**n**^ (µ/ml)	% Protection					
	MPH	MPA	MPC	MPM	MPW	INDO
1000	14.19 ± 0.55	22.63 ± 0.18***	7.65 ± 0.34	40.64 ± 0.78***	8.10 ± 0.67	14.04 ± 0.57
2000	49.26 ± 0.79***	33.61 ± 0.40*	13.40 ± 0.50	51.66 ± 2.51***	26.29 ± 1.08	27.79 ± 0.36
3000	54.46 ± 0.34***	51.95 ± 0.34**	18.48 ± 0.67	56.85 ± 0.69***	59.98 ± 1.34***	46.69 ± 0.42
4000	59.95 ± 0.49	53.30 ± 0.47	22.96 ± 0.72	62.19 ± 1.12***	67.99 ± 1.49	63.95 ± 0.49
5000	65.14 ± 0.10	59.87 ± 0.52	26.63 ± 0.69	70.93 ± 3.06	80.50 ± 0.38	83.30 ± 0.46

Values are Mean ± SE (n = 3). *P value* (***): very highly significant; (**): highly significant, (*): significant difference over the standard group (Comparison of ‘r’ value).

### Antimicrobial activity

**Figure 2:**
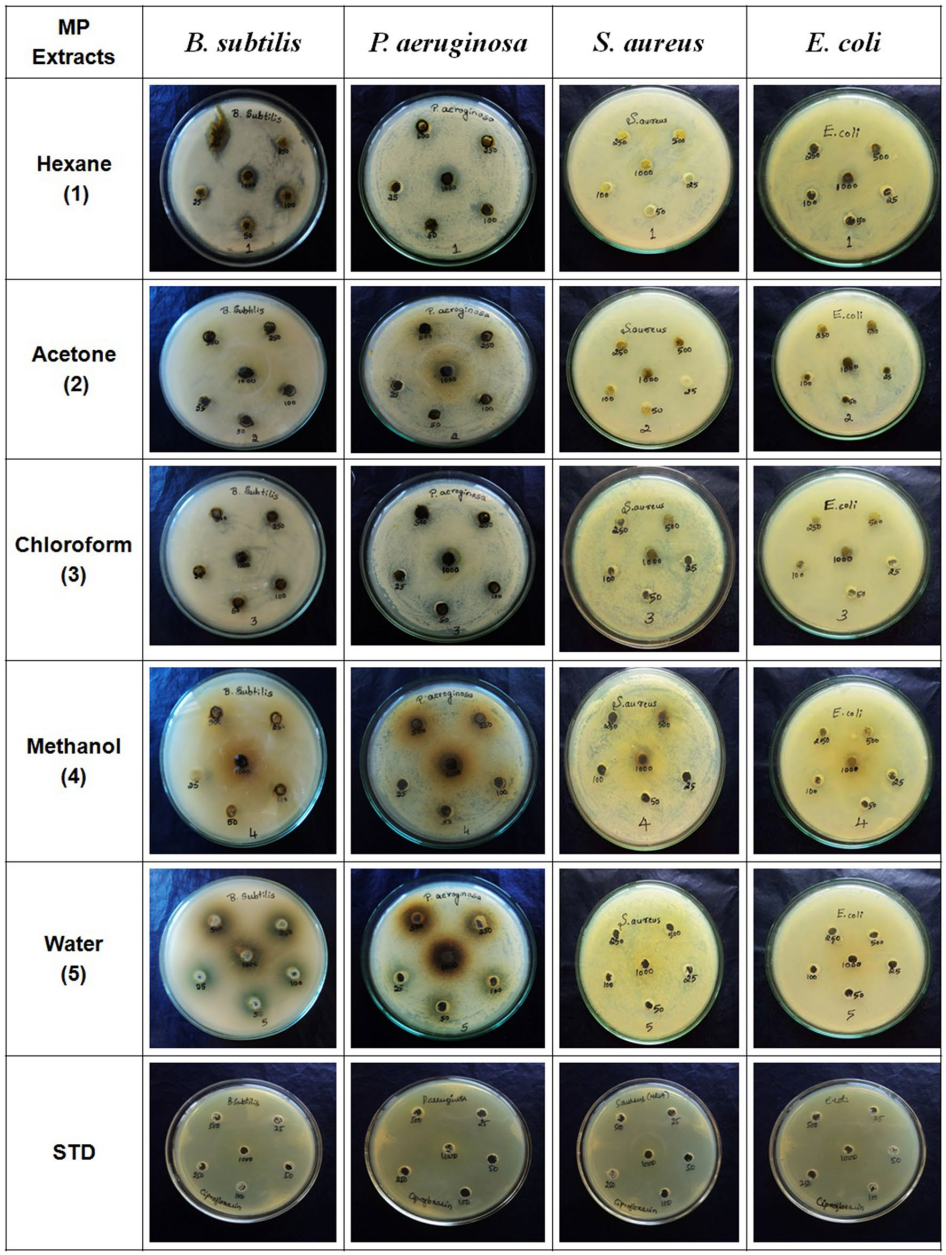
Antibacterial activity of different leaf extracts of *M. paniculata* against *B. subtilis, aeruginosa, S. aureus* and *E. coli.* The drug ciprofloxacin was used as positive controls.

Table 4 presents antimicrobial activity against tested bacterial (*E. coli, S. aureus, P.aeruginosa, B. subtilis)* and fungal (*C. albicans, P. chrysogenum*, *A. niger, A. flavus)* strains using agar well diffusion method (Fig. 2). The growth of *P. aeruginosa* was inhibited by all extracts of *M. paniculat*a. Growth inhibition was observed in standard ciprofloxacin (MIC of 25 µg/mL) against all tested bacterial strains. The MIC for different extracts was as 25 µg/mL for aqueous extract while 250 µg/mL for hexane, methanol and chloroform extracts and 500 µg/mL for acetone extract. The hexane extract displayed good inhibition activity against all the bacterial strains as compared to other tested extracts. All extracts were found inactive against fungi as we did not observe any zone of inhibition even after repeated experiments.

**Table 4:** Anti-microbial activity of various crude extracts of *M. paniculata.*

Crude extract	Mean diameter^1^ (mm) of inhibition zone of bacterial strains^2^							
	Ec		Bs		Pa		Sa	
	Inhibition zone	MIC µg/ml	Inhibition zone	MIC µg/ml	Inhibition zone	MIC µg/ml	Inhibition zone	MIC µg/ml
MPH	3.3***	100	3.6***	50	4.3***	250	3.6***	1000
MPA	3***	500	NF	NF	3***	500	NF	NF
MPC	5.3***	500	4***	1000	3.3***	250	NF	NF
MPM	5.6***	1000	NF	NF	4***	250	3.3***	500
MPW	NF	NF	4***	1000	3***	25	NF	NF
Ciprofloxacin	25.3	25	20.3	25	29.6	25	24.3	25

Crude extract	Mean diameter^1^ (mm) of inhibition zone of fungal strains^2^							
	Ca	Pc	An	Af				
	Inhibition zone	Inhibition zone	Inhibition zone	Inhibition zone				
MPH	NF	NF	NF	NF				
MPA	NF	NF	NF	NF				
MPC	NF	NF	NF	NF				
MPM	NF	NF	NF	NF				
MPW	NF	NF	NF	NF				
Ciprofloxacin	-	-	-	-				

^1^*n* = *3* Standard error was < 15% of mean in all cases, *P value* (***) represents very highly significant difference over the standard group. ^2^Ec: *Escherichia coli*, Bs: *Bacillus subtilis*, Pa: *Pseudomonas aeruginosa,* Sa: *Staphylococcus aureus ,* Ca: *Candida albicans,* Pc: *Penicillium chrysogenum*, An: *Aspergillus niger *and Af: *Aspergillus flavus*. ^3^NF: Not found any zone of inhibition.

### Fractionization of hexane extract

13 fractions were collected initially using column chromatography. The results of TLC showed that F1- F3 contain single spot and were eluted using hexane and dichloromethane (5:5) as the solvent. The fractions were pooled and renamed as PC1. The F9 & 10 showed similar spots and were pooled as PC3. Further F11-13 had similar spots and was renamed as PC4. The F4 to F8 displayed 3 spots on TLC (2 spots in UV and 1 spot in Vis) which were concentrated and further allowed for column chromatography separation using mixture of hexane and acetone as solvent. From the repeated chromatography we obtained fraction PC2 (in visible region), PC11, and PC22 (in UV region). Finally, 6 pooled fractions were obtained, named as PC1, PC2, PC3, PC4, PC11, and PC22, and were used for further antibacterial activities.

### Antibacterial activity

**Figure 3:**
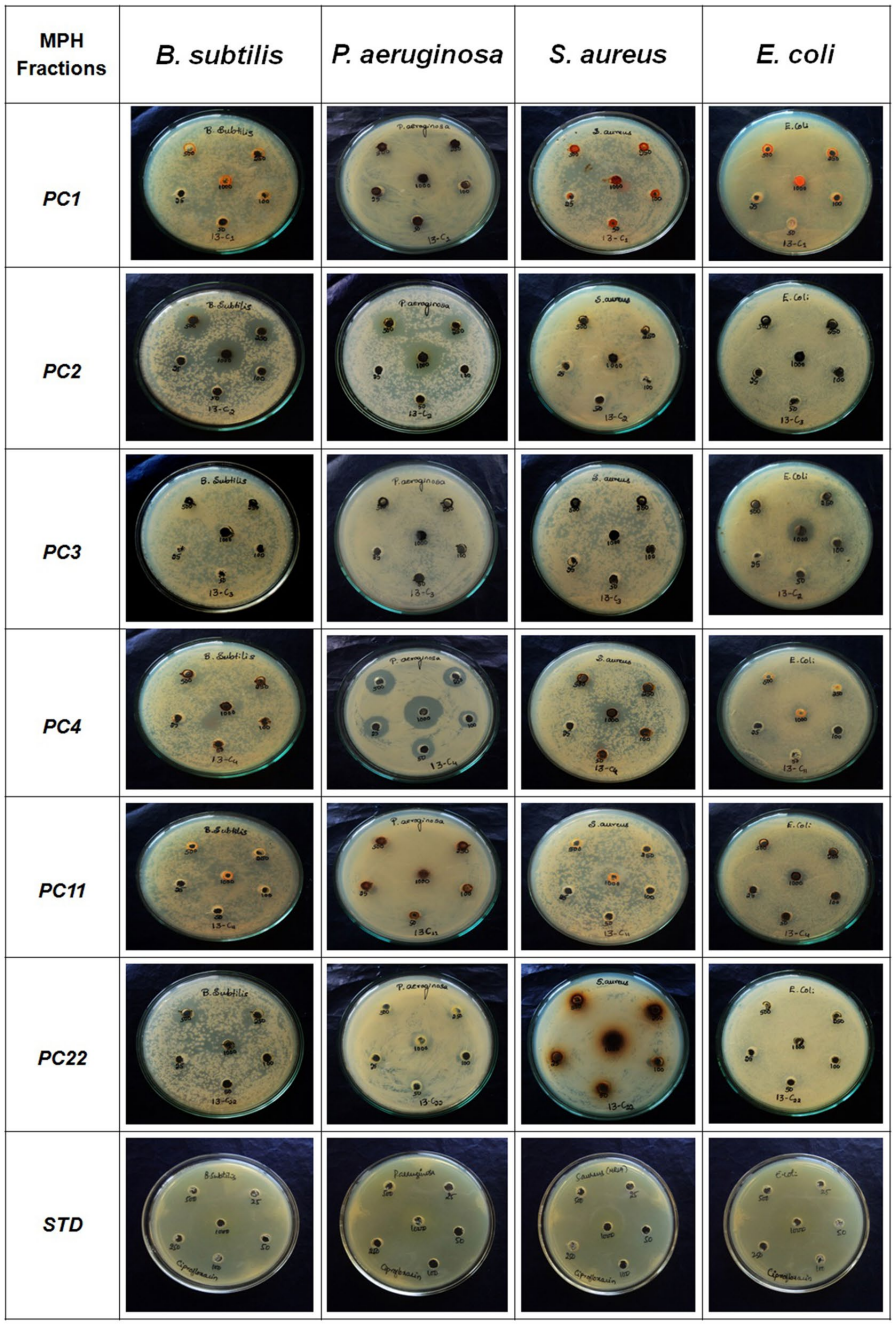
Antibacterial activity of hexane fractions of *M. paniculata* leaves against *B. subtilis, P.aeruginosa, S. aureus* and *E. coli.* PC1, PC2, PC3, PC4, PC11, PC22 represents different collected fractions and the drug ciprofloxacin were used as standard.

Table 5 presents antimicrobial activity against selected multi resistance bacteria (*E. coli, S. aureus, P. aeruginosa, B. subtilis)* using fractions obtained from column chromatography. Growth inhibition was observed in standard ciprofloxacin (MIC of 25 µg/mL) against all the tested bacterial strains. The *B. subtilis* and *P. aeruginosa* were inhibited by all fractions of hexane extract, out of which PC4 showed MIC of 250 and 25 µg/mL respectively (Fig. 3).

**Table 5:** Anti bacterial activity of fractions of *M. paniculata* hexane extracts.

Fractions	Mean diameter^1^ (mm) of inhibition zone of bacterial strains							
	Ec		Bs		Pa		Sa	
	Inhibition zone	MIC µg/ml	Inhibition zone	MIC µg/ml	Inhibition zone	MIC µg/ml	Inhibition zone	MIC µg/ml
PC1	NF	NF	3***	1000	3***	250	3***	1000
PC2	3***	250	3.3***	25	9.6***	250	NF	NF
PC3	NF	NF	2.3***	1000	3***	1000	NF	NF
PC4	3***	250	3.3***	250	6.3***	25	3***	250
PC11	2.3***	500	3***	1000	4.6***	500	3.3***	1000
PC22	NF	NF	3***	250	3***	250	3.6***	250
Ciprofloxacin	25.3	25	20.3	25	29.6	25	24.3	25

Ec: *Escherichia coli*, Bs: *Bacillus subtilis*, Pa: *Pseudomonas aeruginosa,* Sa: *Staphylococcus aureus.* NF: Not found any zone of inhibition. ^1^*n* = *3* Standard error was < 15% of mean in all cases. *P value* (***) represents very highly significant difference over the standard group.

### GC–MS/MS of PC4 fraction of Hexane extract

**Figure 4:**
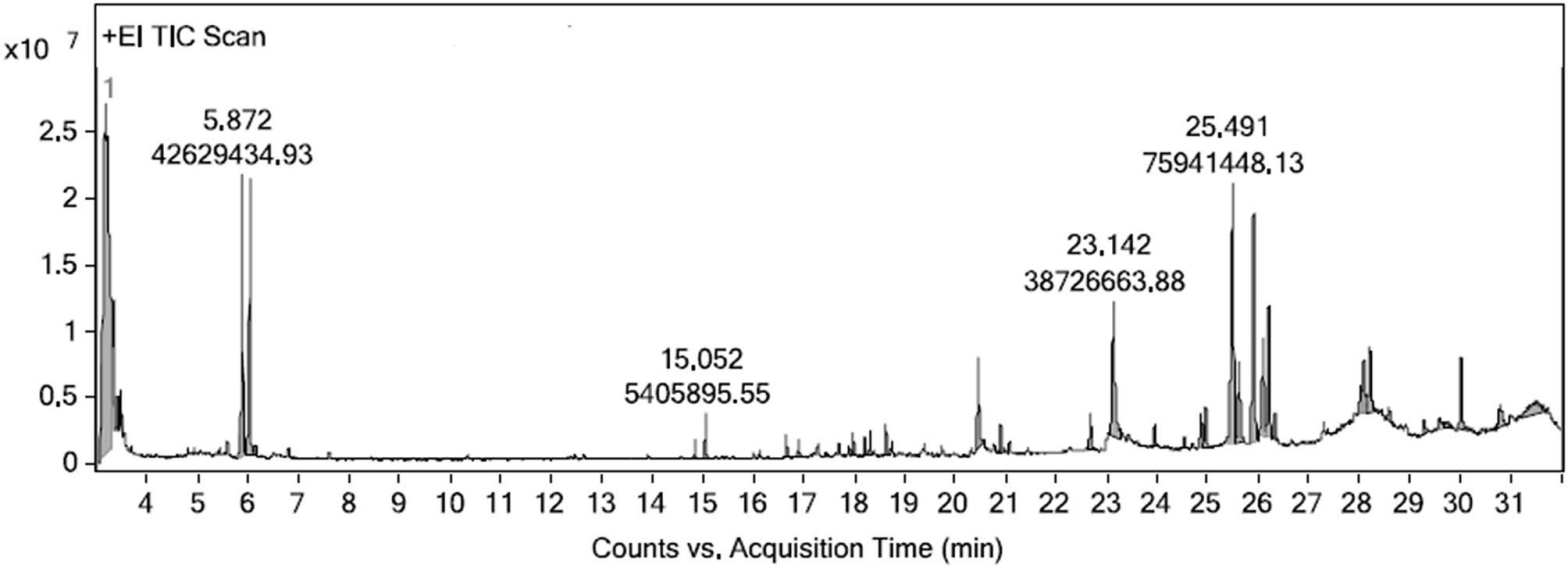
GC–MS/MS chromatogram of PC4 fraction (Acetone: methanol) of *M. paniculata* Hexane extract. The peaks represent the active compounds present in the PC4 fraction.

The PC4 fraction from the hexane extract exhibited significant antibacterial activity was subjected to analysis by GC–MS/MS technique. The major antibacterial components found in the hexane fractions were Cyclohexane (40.11%) and 3-(6-Methoxy-3-methyl-2-benzofuranyl)Cyclohexanone (13.68%) is shown in Table 6 and Fig. 4. The supplementary figure (Fig. S3) shows mass spectrum of major components identified through GC–MS/MS analysis.

**Table 6:** The major components found in the PC4 fraction (Acetone : Methanol) of Hexane extract.

Si No	Area	Area%	Compound Name	RT	Score (Lib)	Mol. Wt	Mol. Formula
1	16,972,971.39	3.05	2-Propenoic Acid, 2-Methyl-(Tetrahydro-2-Furanyl)Methyl Ester	3.081	86.51	170.21	C_9_H_14_O_3_
2	222,543,567	40.11	Cyclohexane	3.169	92.86	84.16	C_6_H_12_
3	12,736,050.36	2.29	Heptane, 2,3-dimethyl	3.324	82.69	126.24	C_9_H_20_
4	42,629,434.93	7.68	Hydroperoxide, 1-ethylbutyl	5.872	85.51	118.17	C_6_H_14_O_2_
5	40,284,396.03	7.26	Hydroperoxide, 1-ethylbutyl	6.027	88.62	118.17	C_6_H_14_O_2_
6	38,726,663.88	6.98	9,12,15-octadecatrienoic acid, methyl ester,(Z,Z,Z)-	23.142	87.82	292.5	C_19_H_32_O_2_
7	75,941,448.13	13.68	3-(6-Methoxy-3-methyl-2- benzofuranyl) cyclohexanone	25.491	87.97	280.53	C_20_H_40_
8	52,269,994	9.42	4,4′-ethylenebis(2,6-di-tert-butylphenol)	25.908	59.3	438.7	C_30_H_46_O_2_
9	24,304,527.71	4.38	Phenol, 2,4-bis(1,1-dimethylpropyl)-	26.097	62.69	234.37	C_16_H_26_O
10	28,369,126.04	5.11	2H-1-Benzopyran-2-one, 6-(*3*-hydroxy-3-methylbutyl)-7-methoxy-	26.206	56.79	260.28	C_15_H_16_O_4_

## Discussion

Amarkantak is located in an ecologically diverse region in the states of Chhattisgarh and Madhya Pradesh,India. Several tribal and non-tribal communities reside in this area and utilize the forest produce for their livelihood and other needs. Being biodiversity rich, Amarkantak is a potential source of hundreds of medicinal plants that are being utilized by the tribal communities for the treatment of various health problems. *M. paniculata* is used by them for treatment of tuberculosis, malaria, wound healing, and as an antidote for snakebite. Therefore, *M. paniculata* leaves were selected for investigation of antioxidant, membrane stabilizing, and antimicrobial properties stemming from phytochemical features. Phytochemical screening determines the presence of alkaloids, flavonoids, steroids, tannins, saponins and glycosides that are known to have protective or disease preventive properties. Several phytochemicals play vital roles in alleviating the several health problems like malaria, asthma, cancer, arthritis, jaundice, diabetes, dengue, diarrhea, dysentery, and microbial infections^45^. In the current study, secondary metabolites such as alkaloids, flavonoids, steroids andtannins were present in majority of extracts, corroborated by earlier studies^46^ Alkaloids, tannins flavonoids and other metabolites are reported to have antioxidant, antifungal, anticancer, antiviral, anti-inflammatory and antiophidic activities^47^. Alkaloids have been the basis of several antibacterial drugs and serve as scaffolds for important antibacterial drugs such as metronidazole, quinolones, linezolid, and trimethoprim^48,49^. More than 8000 flavonoids have been reported from different plants species^50^. That play vital role in stimulation, protection, flavoring, communication, and pigmentation^51^. Flavonoids are rich source of antioxidants mainly due to their free radical scavenging activity by the transfer of an H atom or of a single electron to the radical stabilizing it or due to their metal chelating activity^52,53^. The flavonoids are known to possess anti-allergic, hepatoprotective anticancer, antibacterial, anti-inflammatory, anti-diabetic, and anti-viral properties^54–58^. Saponins, a vast group of compounds are characterized by their foam forming and detergent properties^59–61^. They are known to have several biological properties like antimicrobial, immunomodulatory, anti-malarial, anti-allergic, anti-diabetic, insecticidal, and anti-inflammatory^62–66^. The tannins are water soluble polyphenolic compounds synthesized by plants^62^ and are responsible to protect plants against herbivores and insects^67,68^. Tannins have also been reported to have anti-cancer, anti mutagenic, anti-oxidant, antibacterial, anti-viral, anti-tumor, anti-inflammatory effects and wound healing capabilities^68–73^. The presence of all these phytochemicals makes it understandable why *M. paniculuta* leaves are a prominent medicine for treatment of various diseases within tribal communities.

Here, the total phenolic content was present in high concentrations in methanolic extract followed by acetone, water, chloroform, and hexane while the total flavonoid content was highest in methanol extract followed by water, acetone, hexane and chloroform. Antioxidant activity of flavonoids is due to their ability to reduce free radical formation and to scavenge free radicals. Previously, *Zhang *et al. (2011) reported the presence of seventy polymethoxylated flavonoids (PMF) in the leaves extract of *M. paniculata*^74^; which are responsible for a number of biological activities including antioxidant^75,76^ and antimicrobial properties^77,78^. Phenolic compounds are good antioxidants as their hydroxyl groups can directly contribute to antioxidant action leading to a wide range of biological effects including antibacterial, anti-inflammatory, anti-allergic, hepato-protective, antithrombotic, antiviral, anticarcinogenic, and vasodilatory actions^79^. Some phenolic compounds can stimulate the synthesis of endogenous antioxidant molecules in the cell^80^. The results of antioxidant activities correlated well with the presence of phenolic and flavonoids in different extracts.

Significant concentration-dependent inhibition of ABTS free radicals was observed in all the tested extracts. The IC_50_ value observed for acetone extract was less than the standard ascorbic acid, indicating the strong ability of the extracts to act as ABTS and H_2_O_2_ scavenger. Significant dose-dependent H_2_O_2_ scavenging activities were observed in all the extracts analyzed. The lowest IC_50_ (509.84 μg/mL) was observed for the hexane extract that was lower than that of ascorbic acid (528.01 μg/mL) although it was observed that at low concentrations ascorbic acid was found to be more efficient. The reducing capacity of antioxidant is mainly because of their electron transfer property for instance polyphenols and flavonoids ^81^. The FRAP assay is a relatively simple, quick, and inexpensive direct method of measuring the combined antioxidant activity of reductive antioxidants in a test sample ^82^. The higher values shows, higher antioxidant property of extracts. Our study clearly indicated that the methanolic extract of the leaves exhibits high scavenging capacity, demonstrating that the high antioxidant capacity of the methanol extract correlates with the total content of phenolic and flavonoid content (i.e., higher the absorbance shows higher antioxidant properties). The present study also demonstrated the results that total antioxidant reducing power of compound/extract is depending on the amount of phenolic and flavonoid content. The comparison between the results of total phenolic content and reducing power assay is found to be in similar order: MPM > MPW > MPA > MPH > MPC. Whereas the results of total flavonoid content and FRAP values found to be in order of: MPM > MPA > MPW > MPC > MPH. Many studies have demonstrated a strong correlation between the phenolic content and reducing property of compounds. The redox properties of phenolic are major reason for the antioxidant property of an extract^83,84^.

Low-grade inflammatory state is correlated with various disorders and chronic health conditions, such as obesity, diabetes, cancer, and cardiovascular diseases. Living tissues respond to injury, infection or irritation by releasing inflammatory lysosomal enzymes that may damage macromolecules and cause lipid peroxidation of membranes. Plant extracts can be utilized for stabilization of lysosomal membranes or control of major pro-inflammatory mediators by inhibiting the release of lysosomal constituents of activated neutrophil such as bactericidal enzymes and proteases. Human red blood cell (HRBC) or erythrocyte membranes are analogous to lysosomal membranes and have been used in stabilization assays. The antioxidant activity of the *M. paniculata* extracts correlated well with the RBC membrane stabilization properties. At the concentrations of 4000–5000 µg/mL, 60–80% protection was observed, which was comparable to the standard drug indomethacin in RBC hemolysis with water and methanol extract. Non-steroidal anti-inflammatory drugs (NSAIDs), such as indomethacin, acts by inhibiting the enzyme cyclooxygenase, but the use of NSAIDs is questionable due to the emerging evidence suggesting a high risk of acute myocardial infarction, stroke, heart failure, renal failure, and arterial hypertension^85^. Several medicinal plants have been reported to have phytoconstituents such steroids, flavonoids, alkaloids, polyphenols, glycosides, terpenoids, curcumins, GLA, linear aliphatic alcohols, harpagoside, phenolic diterpenes, which have anti-inflammatory properties with minimal side effects^14^. The results of our study clearly suggest that *M. paniculata* extracts may provide us with some potent compounds for the management of inflammatory conditions. The presence of saponins and flavonoids in the methanolic and water extracts also support the current findings as they have been known to have profound stabilizing effect on lysosomal membrane^86^. It has been reported that flavonoids isolated from *M. paniculata* displayed anti-inflammatory activity by inhibition of NO and IL-6 production^87^. The development of flavonoid based phytotherapeutic anti-inflammatory agents, Acheflan and Daflon, gives us hope for the development of drugs without adverse effects^85^. Novel safe and promising antimicrobial agents are urgently required to fight against drug-resistant bacterial and fungal strains. Flavonoids are known to inhibit microbial growth by inhibition of nucleic acid synthesis, cytoplasmic membrane function and energy metabolism pathways^88^. The antimicrobial properties of phenolic compounds have been reported due to the alteration of cell membrane permeability leading to the uncoupling of oxidative phosphorylation, inhibition of active transport and cytoplasmic membrane damage that leads to the loss of pool metabolites^89^. Tannins have been shown to possess inhibitory activities against Methicillin-resistant *Staphylococcus aureus* (MRSA)^90^ or bacteria, yeast and fungi^91^. In the current study all the extracts of *M. paniculata* displayed some level of inhibition of bacterial growth, but no antifungal activity was observed displaying its potency for the development novel antibiotics. Here, both the Gram negative and Gram positive bacteria were inhibited by the extracts. However, the hexane extract exhibited growth inhibition against all tested bacterial strains with a minimum observed MIC of 50 µg/mL against *B. subtilis* or MIC 250 µg/mL against *P. aeruginosa*. Fractionization of hexane extract yielded six fractions that revealed promising antibacterial potency. The *P. aeruginosa* was found to be very sensitive to the fractions of hexane extract displaying an inhibition zone of 9.6 mm against PC2 fraction, whereas the PC4 fraction exhibited considerable antibacterial activity against all tested strains. *P. aeruginosa*, an opportunistic pathogen, is the leading cause of morbidity and mortality in immune-compromised individuals or cystic fibrosis patients^92^ and displays remarkable antibiotic resistance capacities. It has been listed by WHO as one of the three bacterial species against which there is critical need for the development of new antibiotics^93^. Similarly, methicillin-resistant *S.* *aureus* have been associated with hospitals settings and has emerged as a widespread cause of community infections. The inhibition of both these strains by PC4 predicts it to be a source of novel antibacterial compound/s specifically against *P. aeruginosa* and *S. aureus*.

The GC–MS/MS analysis of PC4 fraction revealed a presence of cyclohexane and 3-(6-Methoxy-3-methyl-2- benzofuranyl) cyclohexanone that might be a reason for the observed antibacterial activity. The cyclohexane and derivates are lipophilic weak acids and possess exceptional nature of mode of action as antibacterial agents. Unlike other cationic antimicrobial compounds, these cyclohexane and derivatives will not interrupt the cytoplasmic membrane instead they block the transport of low molecular weight hydrophobic substances of bacterial cell^94^. The heterocyclic ring compounds, such as benzofuran, is a significant class of compounds obtained. This class of compounds is vital in numerous pharmacological areas and could be a one such reasons for the active biological properties of natural products^95^. The derivatives of benzofuran exhibited many noteworthy activities against viruses, fungi^96,97^ protozoan, tuberculosis^98^ and so on. Therefore, the wide range of biological importance coupled with this scaffold has resulted in the cyclohexane and benzofuran ring systems being considered as a privileged structure. Much attention is warranted for cyclohexane derivatives and benzofuran-based medicinal agents, hence it is important that research and development on these compounds be increased across a wide range of medicinal areas. Efforts have been invested in the past several decades to develop more effective and less toxic agents to treat many infectious and resistant strains of microbes. Cyclohexane and benzofuran based compounds as microbial agents could be a promising, as they possess structural diversity and excellent therapeutic potency. Our antibacterial activity results coupled with previous studies emphasizes the importance of using plants as critical therapeutic agents.

## Conclusion

The current study illustrates that the *M. paniculata* leaves extracts possess potent antioxidant activity and could stabilize the human RBC membranes in a dose-dependent manner, thus providing novel anti-inflammatory compounds. A preliminary chemical examination of different extracts reveals the presence of flavonoids, alkaloids, tannins, and polyphenols that might be responsible for the antimicrobial, antioxidant, RBC membrane stabilization, and anti inflammatory properties. The PC4 is the most potent hexane fraction that was bactericidal against *E. coli, B. subtilis, P. aeruginosa, S. aureus,* and further characterization revealed the presence of cyclohexane and banzofuran derivatives that might be responsible for the observed antimicrobial activity. The overall results of the present study demonstrate that traditionally used plants provide extremely promising prospects compounds for the development of novel anti-microbial drugs ; especially as treatments against multi-drug resistant bacterial strains.

## Materials and method

All the methods were carried out in compliance with relevant guidelines.

### Preparation of extracts

The leaves of *M. paniculata* L. growing in wild were collected from the Amarkantak region of Madhya Pradesh, India, and were identified by Dr. Ravindra Shukla, Department of Botany, Indira Gandhi National Tribal University, Amarkantak, MP (India). A voucher specimen of the plant (SS/DOB/MP13) has been deposited in the Department of Botany for future reference. The leaves were washed under tap water, shade dried and finally grinded it into fine powder form. The extraction was carried out using different solvents (hexane, acetone, chloroform, methanol and water) for approximately 7–8 h using Soxhlet extractor and concentrated using rotary evaporator. The extract was dried and stored in vials at 4^0^C till future use.

### Phytochemical screening

The phytochemical screening of all extracts was carried out according to standard methods as mentioned elsewhere with minor modifications^99–101^.

### Total phenolic content

The total phenolic content was determined for different *M. paniculata* extracts using spectrophotometric methods^102^. Different extracts were taken at concentration 1 mg/mL for analysis. The reaction mixture was prepared (in triplicate) by mixing 0.5 mL of individual extracts, 2.5 mL of 10% Folin-Ciocalteu’s reagent dissolved in water and 2.5 mL 7.5% NaHCO_3_. The samples were incubated for 45mins at 45^0^C and the absorbance was measured at 765 nm using spectrophotometer (Shimadzu UV–Vis spectrophotometer-1800). Based on the measured absorbance, the total phenolic content in the test samples was calculated using the calibration plot (*Y* = *0.00024x* + *0.03510, R2* = *0.9963*) and expressed in terms of Gallic acid equivalent (mg of GA/g of extract).

### Total flavonoids content

The total flavonoid content was performed as per Dowd method with some modifications for the different extracts^103–105^. Different extracts were taken at 1 mg/mL concentration for analysis and reaction mixture prepared by mixing 0.5 mL of extracts, 10% aluminium chloride (0.1 mL), 1 M potassium acetate (0.1 mL) and distilled water (4.3 mL). Reaction mixture was incubated at room temperature for 30 min and absorbance was measured at 510 nm .Quercetin (1 mg/mL) was used for preparing the standard calibration curve. The total flavonoid content in the test samples was calculated using the calibration plot (*Y* = 0.002*x* + 0.158, *R*^2^ = 0.997) and expressed as mg quercetin equivalent (QE)/g of dried plant material.

### Anti oxidant assays

#### ABTS (2,2′-azino-bis(3-ethylbenzothiazoline-6-sulfonic acid) assay

ABTS is a synthetic radical widely used for both the polar and non-polar samples^106^. Mixture of ABTS (14 mM) and potassium per sulfate (4.95 mM) (1:1 v/v) was prepared and allowed to stand overnight at room temperature (RT) in dark. ABTS^+^ was diluted with water to obtain equilibration of absorbance 0.70 (± 0.02) at 734 nm and suitable blank was used without adding test samples. 50 mg/mL stock solutions of plant extracts were prepared and different concentrations used for assay. The following formula was used to calculate percentage of inhibition.

$$Percentage \,of \,ABTS\, inhibition = \left( {A0 - A1/A0} \right) X 100$$

A_0_ –– Absorbance of blank, A_1_ –– Absorbance of Sample.

#### Hydrogen peroxide (H2O_2_) radical scavenging assay

Hydrogen peroxide radical scavenging activity was determined according to previously utilized method^107^. 40 mM H_2_O_2_ solution was prepared in 50 mM phosphate buffer (pH 7.4). 3.4 mL of different concentrations of extracts (1000, 2000, 3000, 4000, and 5000 μg/mL) were added to 0.6 mL of H_2_O_2_ solution. The absorbance was read at 230 nm after 10 min of incubation at RT against a blank solution containing only phosphate buffer without H_2_O_2_.

$$Percentage\, of\, scavenged \,H2O2 = \left( {A0 - A1/A0} \right) X 100$$

A_0_ –– Absorbance of blank, A_1_ –– Absorbance of Sample.

#### Ferric reducing antioxidant power (FRAP) assay

The total antioxidant potency of extracts of *M. paniculata* was estimated using a ferric reducing antioxidant power (FRAP) assay^108^. FRAP reagent (straw yellow color) was prepared by mixing 30 mM acetate buffer, 10 mM TPTZ, 20 mM FeCl_3_ and distilled water. The standard curve of ferrous sulfate (FeSO_4_.7H_2_O) was prepared with concentrations ranging from 0.1, 0.2, 0.4, 0.4, 0.6, 0.8 and 1.0 mM/L. Different concentrations of ascorbic acid and sample (1000, 2000, 3000, 4000, and 5000 μg/mL) were prepared from 1 mg/mL stock solution and 3 mL of FRAP reagent added to each and the tubes were incubated for 30 min in the dark, read absorbance at 593 nm.

#### Reducing power assay (RPA)

The total reducing power of extracts was estimated by the method of Oyaizu et al. (1986)^109^. The different concentrations of plant extracts (1 mL of 1000, 2000, 3000, 4000, and 5000 μg/mL) were mixed with 5 mL of 0.2 M phosphate buffer (pH-6.6), and 5 mL of 1% ferricyanide was added and incubated for 20 min at 50^0^C. After incubation the 10% trichloro acetic acid (TCA) added and centrifuged at 3000 rpm for 10 min. Equal amounts of water and 1 mL of 1% ferric chloride were added to the supernatant, absorbance read at 700 nm. Higher the absorbance value of the reaction mixture indicated better reducing power.

### Membrane stabilizing property

The HRBC membrane stabilization method is mainly used to estimate the anti-inflammatory activity of plant extracts^110^. The fresh blood was collected and mixed with an equal volume of sterilized Alsever’s solution (2% dextrose, 0.8% sodium citrate, 0.5% citric acid, and 0.42% sodium chloride in water). The administration of NSAIDS for 2 weeks before collecting the blood was avoided in sampled participants. The collected blood was further centrifuged at 3000 rpm for 10 min, the pellet (packed cells) was washed three times with isosaline (0.85%, pH 7.2), and finally 10% (v/v) suspension was made with isosaline. To the different concentrations of plant extracts, 1 mL phosphate buffer (0.15 M, pH 7.4), 2 mL hyposaline (0.36%), and 0.5 mL HRBC suspension was added. Standard and control were prepared without addition of the extracts. Indomethacin at different concentrations (1000, 2000, 3000, 4000, and 5000 μg/mL) was used as the standard drug and compared with respective concentrations of plant extract. The reaction mixtures were incubated at 37^0^C for 30 min and centrifuged at 3000 rpm for 10 min. The hemoglobin content in the supernatant was estimated at 560 nm^111^.

The percentage hemolysis was calculated using the following equation:

$$\% Hemolysis = OD \,of\, the\, test \,sample/OD\, of\, the \,control) x 100$$

The percentage of HRBC membrane stabilization was calculated using the following equation:

$$\% Protection = \left[ {100 {-}\left( {OD \,of \,test \,sample {-}OD \,of\, control} \right)} \right] x 100$$

### Antimicrobial assay

Crude extracts were evaluated for their antimicrobial properties against selected bacterial strains (*Escherichia coli* MTCC 1575*, Staphylococcus aureus* MTCC 1144*, Bacillus subtilis* MTCC 2413*, Pseudomonas aeruginosa* MTCC 1688) and fungal strains (*Candida albicans* MTCC 854*, Penicillin chrysogenum* MTCC 1996, *Aspergillus niger* MTCC 872 *and A. flavus* MTCC 1883).Standard drugs Amphotericin and Ciprofloxacin were used as positive controls.

#### Agar diffusion method

The stock cultures of bacteria were revived by inoculating in broth media and grown at 37ºC for 18 h. The agar plates were prepared, after solidification the 100 μl (10^–4^ cfu) 18 h old culture inoculated and evenly spread. After 20 min, the wells were filled with test compounds at different concentrations (1000, 500, 250, 100, 50, 25 µg/mL) kept for incubation at 37ºC for of 24 h. The diameter of inhibition zones measured and noted as described earlier^112,113^. Minimum inhibitory concentration (MIC) was calculated using the same plate.

### Column chromatography of hexane extract

The most active crude extract (hexane extract) was allowed to separate using gravitation column chromatography. The slurry was prepared by mixing 500 g of absorbent (silica gel 60–120 mesh size) in n-hexane and stirred well to remove bubbles then poured in to glass column. The sample to be loaded on column was prepared by dissolving 5 g of extracting 25 ml of hexane and 20 g of silica. In Table S3, we review the ratio of solvents and fractions obtained in the column chromatography. Each fraction was spotted on activated TLC plate along with extract spot and mobile phase [Hexane: acetone (3:1)] used. The fractions showing more than one spot were concentrated and allowed for further purification using only different solvent mixtures (Hexane and Acetone). The column chromatography was repeated as the eluent system to obtain a single spots on TLC.

### Antibacterial activity

The different fractions collected from the hexane extract were further evaluated for antibacterial activity as mentioned earlier and the zones of inhibition were recorded.

### GC–MS/MS analysis

The GC–MS/MS analysis of PC4 fraction of *M. paniculata* hexane extract was carried out on Agilent technologies model 7890A GC coupled with a mass detector 5975C MS system. The Analytic column was Agilent J&W non polar column DB-5MS ((5%-Phenyl)-methylpolysiloxane, 30 m × 0.25 mm, ID 1.8 micron thickness). Flow rate of helium gas is 1.3 ml/min, used to separate components. The different GC conditions were standardized as follows: injector parameters were injection volume 1 μL under split of 3:1, while injector temperature was set at 280 °C (mass analyzer). During GC extraction the program of oven temperature was 1 min at 75 °C, increased to a temperature of 300 °C at a rate of 30 °C/min for 2 min; inlet and transfer line temperature was 250 °C and 290° C respectively. Mass spectra were taken at an ionization mode with an electron impact at 70 eV. Interpretation of mass spectrum GC–MS/MS analysis was done by matching list of known compound’s spectrum with Agilent’s GC/MS Chemstation, NIST MS Library and NIST’s Automated Mass Spectral Deconvolution and Identification software^114^.

## Statistical analysis

All data were expressed as mean ± SE (n = 3) . Comparison of mean values between various extracts of *M. paniculata* was performed by one way-ANOVA, correlation coefficient (r), and coefficient of determination (r2) calculated using prism 8.0.1(244). The criterion of evaluating statistical significance was as follows: P value < 0.033 was considered significant and marked as *, P < 0.002 as highly significant and marked as **, P < 0.001 was very highly significant and marked as ***.

### Ethics approval and consent to participate

All procedures performed in studies involving human participants were in accordance with the ethical standards of the Institutional Ethics Committee of IGNTU (Approval no. IGNTU/IEC/03/2019) and with the 1964 Helsinki declaration and its later amendments. Researcher’s blood was used for the membrane stabilizing assay and no external participant was enrolled for the study.

## Supplementary Information

**Figure S1**: ABTS radical scavenging activity of different concentrations of *M. paniculata* leaf extracts.** Figure S2**: H2O2 radical scavenging activity of different concentrations of M. paniculata leaf extracts. **Figure S3**. Mass spectrum of major components identified through GC-MSMS analysis.**Table S1**: Phytoconstituents screening of M. paniculata leaf extracts. **Table S2**: Pearson’s correlation coefficient (r) of dose dependent correlation between concentrations versus various extract with inhibition of hemolysis. **Table S3**: Gradient solvent system used in the column-chromatography for the isolation of bioactive molecules from Hexane extract of M. paniculata

## Data Availability

The data can be accessed/shared to the public.

## Abbreviations

ABTS: 2,2′-Azino-bis(3-ethylbenzothiazoline-6-sulfonic acid)
ANOVA: Analysis of variance
FRAP: Ferric reducing antioxidant power
GC–MS/MS: Gas chromatography mass spectroscopy
HRBC: Human red blood cell
H_2_O_2_: Hydrogen peroxide
MIC: Minimum inhibitory concentration
MRSA: Methicillin-resistant *Staphylococcus aureus*
NSAIDs: Non-steroidal anti-inflammatory drugs
NIST: National Institute of Standards and Technology
PC fraction: Pooled concentrated fraction
RPA: Reducing power assay
TCA: Trichloro acetic acid
TLC: Thin layer chromatography
WHO: World health organization
