# Supplementary material for: Chemical profiling, in vitro antioxidant, membrane stabilizing and antimicrobial properties of wild growing Murraya paniculata from Amarkantak (M.P.)
Source: Sci Rep. 2021 May 7;11:9691. doi: 10.1038/s41598-021-87404-7 (PMC8105327; doi:10.1038/s41598-021-87404-7)
Supplement: Supplementary file 1 — Figure S1: ABTS radical scavenging activity of different concentrations of M. paniculata leaf extracts. Figure S2: H2O2 radical scavenging activity of different concentrations of M. paniculata leaf extracts. Figure S3. Mass spectrum of major components identified through GC-MSMS analysis.Table S1: Phytoconstituents screening of M. paniculata leaf extracts. Table S2: Pearson’s correlation coefficient (r) of dose dependent correlation between concentrations versus various extract with inhibition of hemolysis. Table S3: Gradient solvent system used in the column-chromatography for the isolation of bioactive molecules from Hexane extract of M. paniculata [file 41598_2021_87404_MOESM1_ESM.docx]

**Chemical Profiling, *In Vitro* Antioxidant, Membrane Stabilizing and Antimicrobial Properties of Wild growing *Murraya paniculata* from Amarkantak (M.P)**

**Shruti Sonter, Shringika Mishra, Manish Kumar Dwivedi and Prashant Kumar Singh***

Department of Biotechnology, Indira Gandhi National Tribal University, Amarkantak,

(Madhya Pradesh) India

Shruti Sonter, Research Scholar,

Department of Biotechnology, Indira Gandhi National Tribal University, Anuppur, 484887, Madhya Pradesh, India.

E-mail: [shrutiasonter07@gmail.com](mailto:shrutiasonter07@gmail.com)

Shringika Mishra, Research Scholar,

Department of Biotechnology, Indira Gandhi National Tribal University, Anuppur, 484887, Madhya Pradesh, India.

E-mail: [shringik](mailto:shringika99@gmail.com)[a99@gmail.com](mailto:a99@gmail.com)

Manish Kumar Dwivedi, Research Scholar

Department of Biotechnology, Indira Gandhi National Tribal University,

Anuppur, 484887, Madhya Pradesh, India.

E-mail: [dwivedi.manish55@gmail.com](mailto:dwivedi.manish55@gmail.com)

^*^Corresponding author:

Prashant Kumar Singh, PhD

Assistant Professor

Department of Biotechnology, Indira Gandhi National Tribal University,

Anuppur, 484887, Madhya Pradesh, India.

Tel: +91-9179122557

E-mail: [prashant.singh@igntu.ac.in](mailto:prashant.singh@igntu.ac.in)

ORCID ID: 0000-0002-6704-277X

**Table S1. Phytoconstituents screening of *M. paniculata* leaf extracts**

| **Phytoconstituents** | **Presence/Absence in *M. paniculata*** | | | | |
| --- | --- | --- | --- | --- | --- |
|  | **MPH** | **MPA** | **MPC** | **MPM** | **MPW** |
| **Alkaloids** | - | + | + | + | + |
| **Flavonoids** | + | + | - | + | + |
| **Steroids** | - | + | - | + | + |
| **Tannins** | + | + | - | - | + |
| **Phlobatanins** | - | - | - | - | - |
| **Terpenoids** | - | - | - | - | - |
| **Saponins** | - | - | - | + | + |
| **Glycosides** | - | + | - | - | - |

**‘+’**: Represents presence of phytoconstituents, **‘-’:** Represents absence of phytoconstituents

**MPH:** *M. paniculata* hexane, **MPA**: *M. paniculata* acetone, **MPC**: *M. paniculata* chloroform, **MPM:** *M. paniculata* methanol, **MPW**: *M. paniculata* water

Table S2. Pearson’s correlation coefficient (r) of dose dependent correlation between concentrations versus various extract with inhibition of hemolysis.

| **Treatment** | **r** | ***P value*** |
| --- | --- | --- |
| Indomethacin v/s Concentration | 0.9985 | <0.001^***^ |
| MPH v/s Concentration | 0.8843 | 0.05^*^ |
| MPA v/s Concentration | 0.9582 | 0.01^*^ |
| MPC v/s Concentration | 0.9964 | <0.001^***^ |
| MPM v/s Concentration | 0.9903 | 0.001^**^ |
| MPW v/s Concentration | 0.9747 | 0.005^**^ |

**^***^**Very highly significant**; ^**^** highly significant;  **^*^**significant

Table S3. Gradient solvent system used in the column-chromatography for the isolation of bioactive molecules from Hexane extract of *M. paniculata*

| **Solvent system** | **Ratio** | **Volume**  (ml) | **Elutes** | **Pooled fractions** | | **Rf** | **% Yield** (wt/wt) |
| --- | --- | --- | --- | --- | --- | --- | --- |
| Hexane | 100% | 50 | 1 | **PC1** | | 0.95 | 26 |
| Hexane: Dichloromethane | 9:1 | 20 | 2 |  |  |  |  |
| Hexane: Dichloromethane | 5:5 | 20 | 3 |  |  |  |  |
| Hexane: Dichloromethane | 1:9 | 20 | 4 | 3 spots separated from repeated chromatography as | **PC11** | 0.57 | 6.8 |
| chloromethane | 100% | 20 | 5 |  |  |  |  |
| Dichloromethane: Acetone | 9:1 | 20 | 6 |  | **PC2** | 0.42 | 6 |
| Dichloromethane: Acetone | 5:5 | 20 | 7 |  | **PC22** | 0.38 | 8.4 |
| Dichloromethane: Acetone | 1:9 | 20 | 8 |  |  |  |  |
| Acetone | 100% | 20 | 9 | **PC3** | | 0.33 | 16 |
| Acetone: Methanol | 9:1 | 20 | 10 |  |  |  |  |
| Acetone: Methanol | 5:5 | 20 | 11 | **PC4** | | 0.23 | 24 |
| Acetone: Methanol | 1:9 | 20 | 12 |  |  |  |  |
| Methanol | 100% | 50 | 13 |  |  |  |  |

**Supplementary Figures**

**
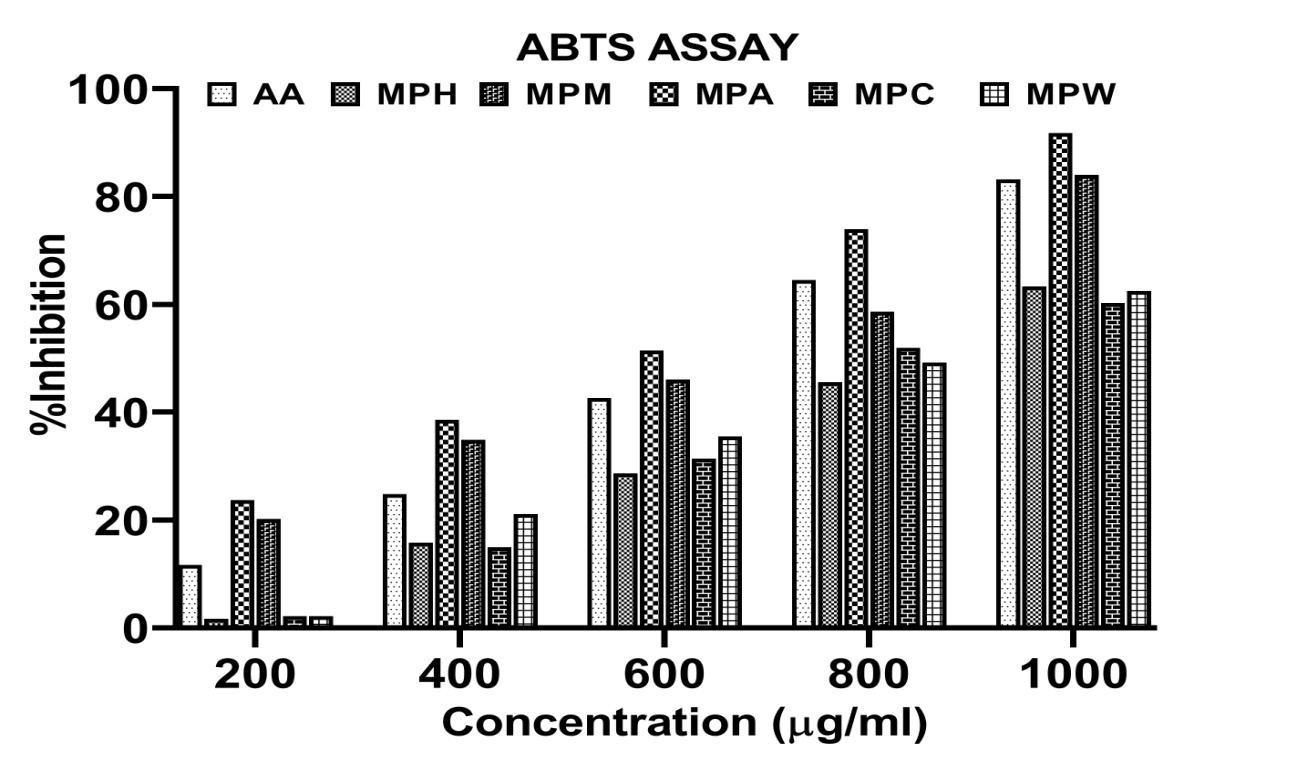
**

Figure S1. ABTS radical scavenging activity of different concentrations of *M. paniculata* leaf extracts. AA: Ascorbic acid, MPH: *M. paniculata* Hexane, MPA: *M. paniculata* Acetone, MPC: *M. paniculata* chloroform, MPM: *M. paniculata* Methanol and MPW: *M. paniculata* water and


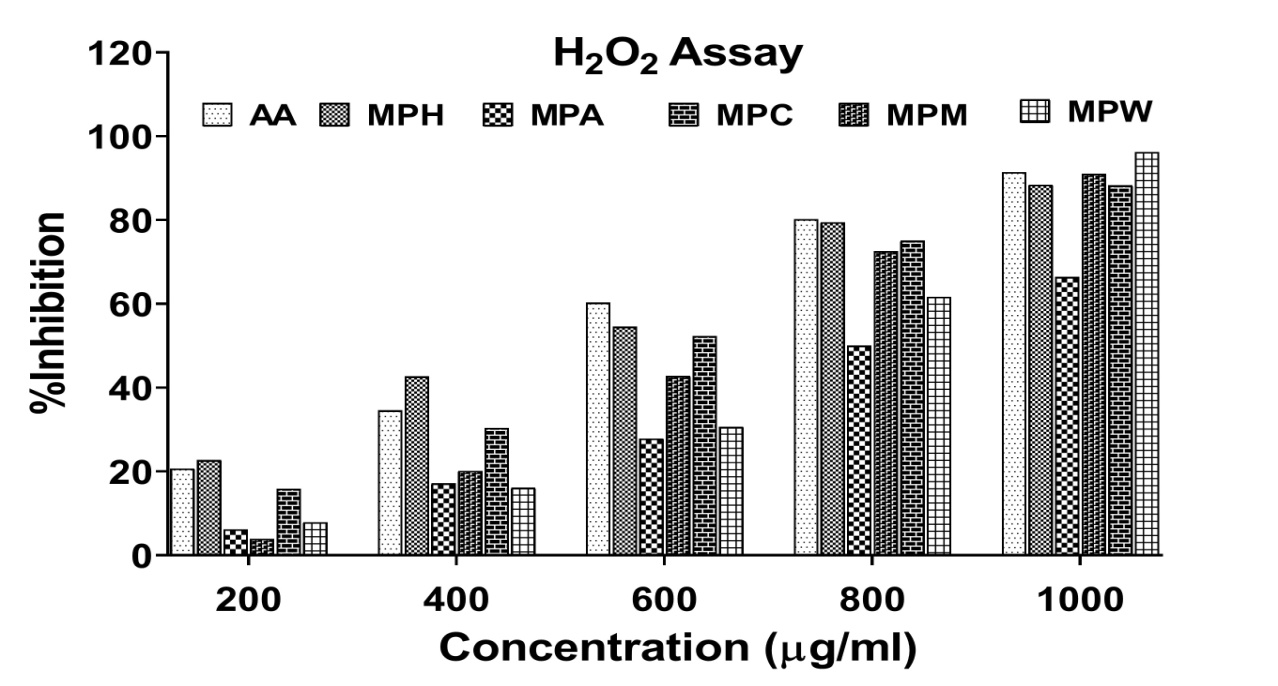


Figure S2. H_2_O_2_ radical scavenging activity of different concentrations of *M. paniculata* leaf extracts. AA: Ascorbic acid, MPH: *M. paniculata* Hexane, MPA: *M. paniculata* Acetone, MPC: *M. paniculata* chloroform, MPM: *M. paniculata* Methanol and MPW: *M. paniculata* water

**
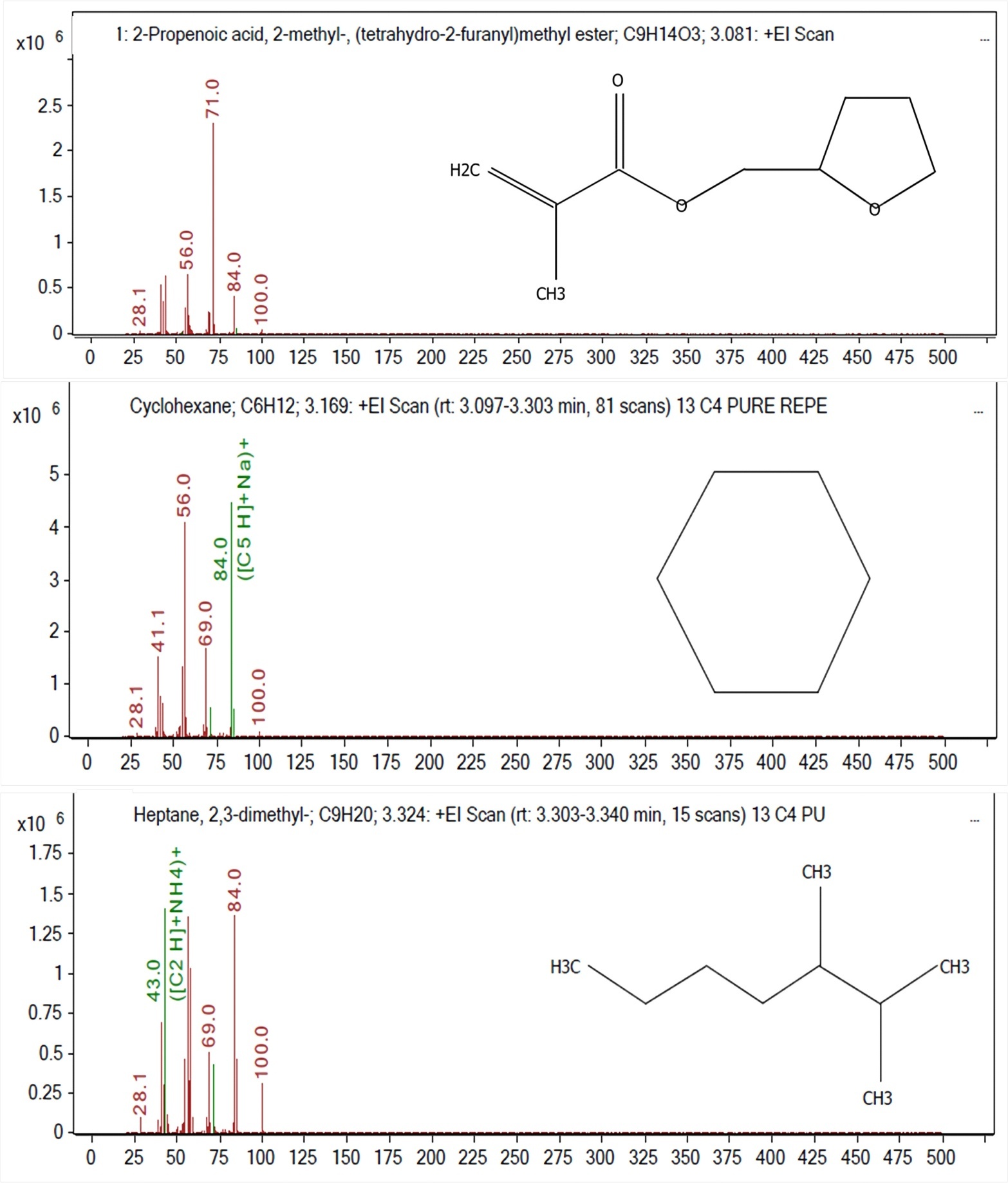
**

**
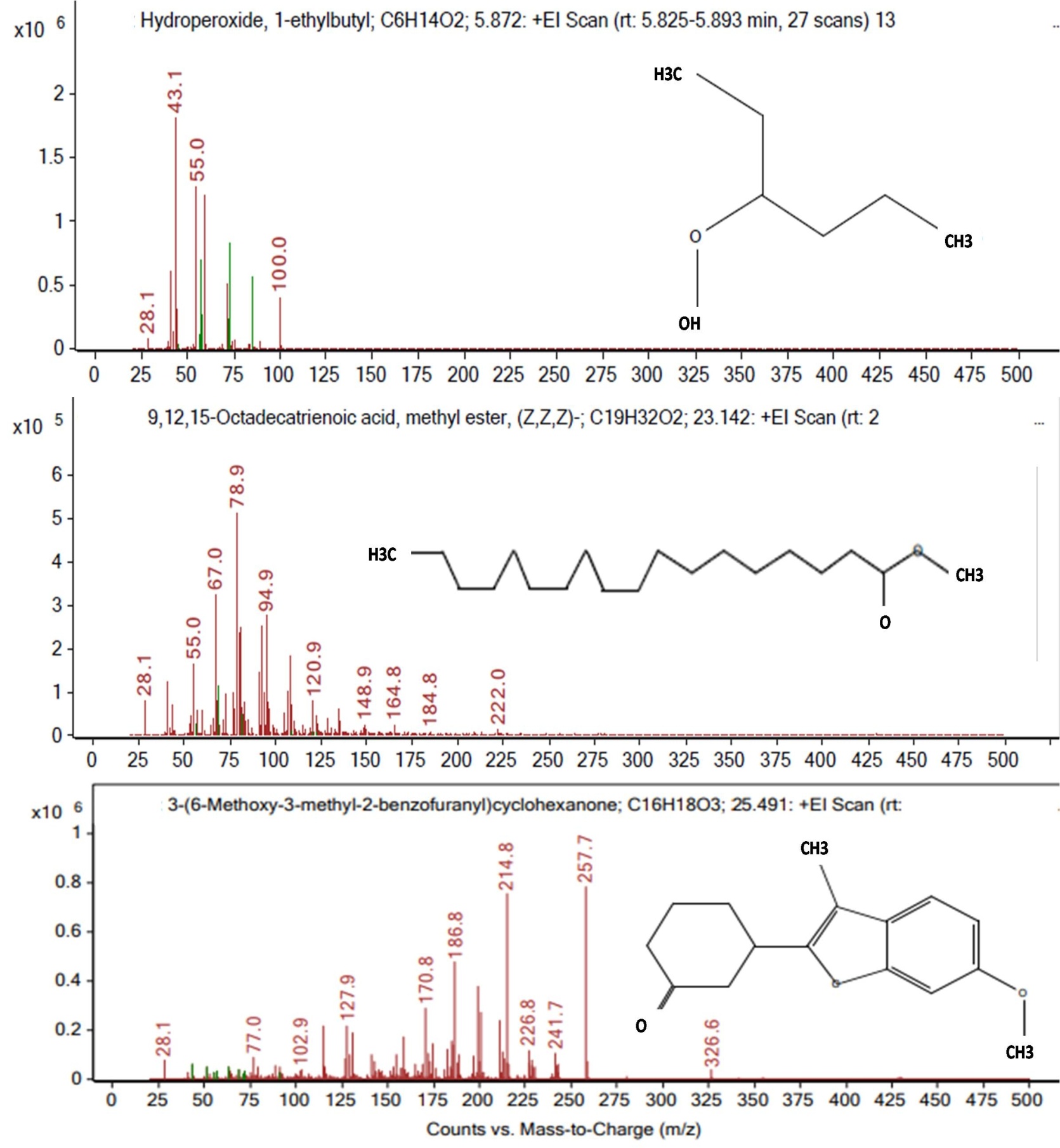

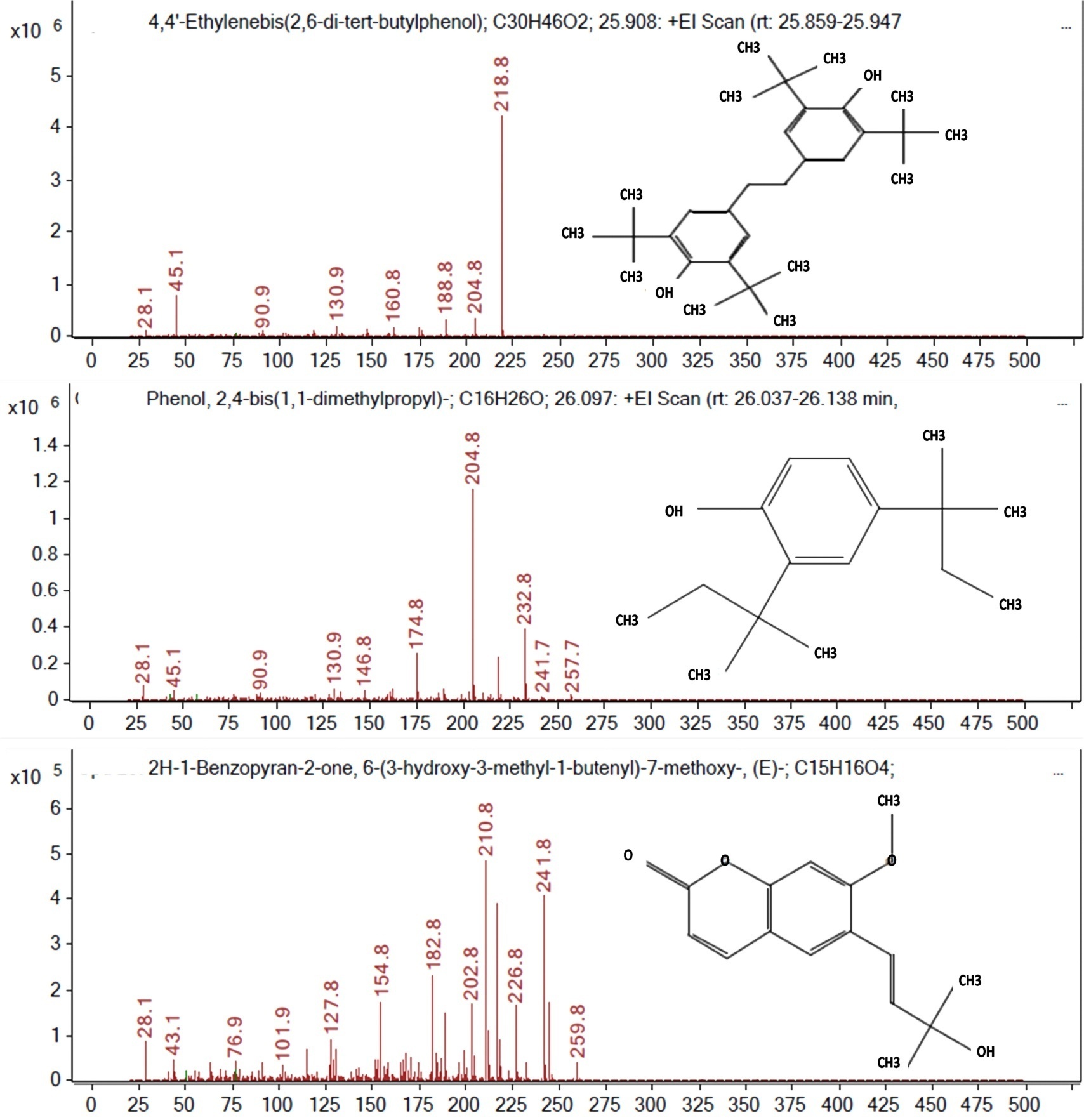
**

**Figure S3. Mass spectrum of major components identified through GC-MS/MS analysis.** The figure shows major 10 compounds MS spectrum of Fraction PC4
